# Femoral head metastases from primary mucinous lung adenocarcinoma with left hip pain: A case report and literature review

**DOI:** 10.3389/fsurg.2022.987627

**Published:** 2022-09-20

**Authors:** Hang Xue, Wu Zhou, Zhenhe Zhang, Adriana C. Panayi, Yuan Xiong, Shuhua Yang, Bobin Mi, Guohui Liu, Xianzhe Liu

**Affiliations:** ^1^ Department of Orthopedics, Tongji Medical College, Union Hospital, Huazhong University of Science and Technology, Wuhan, China; ^2^ Division of Plastic Surgery, Harvard Medical School, Brigham and Women’s Hospital, Boston, MA, United States

**Keywords:** osteonecrosis, bone metastasis, lung adenocarcinoma, atypical symptoms, case report

## Abstract

**Background:**

Primary mucinous lung adenocarcinoma, a subtype of lung adenocarcinoma, is extremely rare. Currently, as there are no specific diagnostic features, it is easy to delay the diagnosis or even to misdiagnose when atypical symptoms are present.

**Case summary:**

This case details a patient with primary mucinous lung adenocarcinoma and metastasis to the femoral head. The sole symptom was left hip pain and the initial diagnosis was isolated femoral head necrosis.

**Conclusions:**

By presenting this rare case report and the experiences learned from it, we hope to assist clinicians to identify bone metastasis cases with non-typical symptoms in order to make the correct diagnosis as soon as possible.

## Introduction

The incidence of bone metastasis from primary lung cancer has been noted to be as high as 30%–40%, and can occur at any time during the progression of lung cancer (1–3). Studies have shown that approximately 70%–90% of patients with bone metastasis from lung cancer are first diagnosed after metastasis (4). The most common site of bone metastasis is the spine, followed by the ribs and pelvis, with some rare reports of mucinous lung adenocarcinoma metastasizing to the testis (5). Mucinous adenocarcinoma is characterized by abundant mucin outside the cancer cells, which is found mostly in the gastrointestinal tract, with a small proportion found in other organs (6). Adenocarcinoma is the most common pathological type seen in bone metastasis of lung cancer, while mucinous lung adenocarcinoma is a rare subtype of lung adenocarcinoma (7, 8). Mucinous lung adenocarcinoma accounts for only 0.24% of all types of lung cancer (9). The prognosis of mucinous lung adenocarcinoma is better than that of other adenocarcinoma subtypes (10). As the incidence of lung cancer is high, and age of diagnosis is gradually decreasing, early detection, diagnosis, and treatment are imperative (11). Especially for lung cancer patients with bone metastasis, due to the atypical symptoms, early and correct diagnosis is very important. In this study we reported a patient with the sole complaint of left hip pain, who was initially diagnosed with femoral head necrosis, but later re-diagnosed with aggressive mucinous adenocarcinoma through clinical, imaging and pathological findings.

## Case presentation

**Figure 1:**
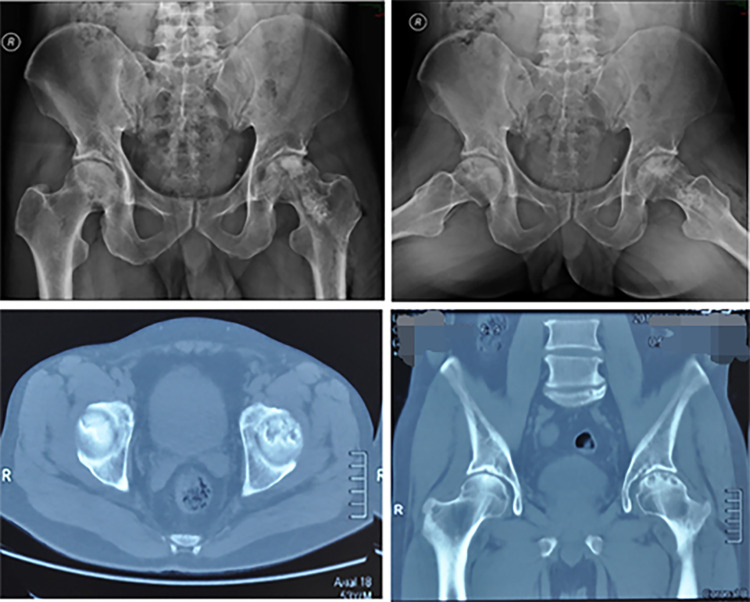
X-ray and CT image results of bilateral femoral heads obtained at the time of the first admission.

A 53-year-old male patient was hospitalized with the chief complaint of left hip pain. The pain had developed one year prior without any obvious cause and was accompanied by mobility problems. The patient underwent physical examination and x-ray radiography at their local hospital and was placed on medication, the details of which were unknown, but the pain had not subsided. When the patient visited our hospital for further evaluation, new x-ray radiographs suggested that the patient had left femoral head necrosis (Figure 1). At the time of admission, the patient’s basic vital signs were within the normal range. During physical examination, the skin overlying the left hip was intact without any rupture, redness, swelling, heat or tenderness. Flexion and extension of the left hip were acceptable, while abduction was limited. Tenderness was identified in the patient’s right rib and back. Given this presentation, our initial diagnosis was left femoral head necrosis with concomitant intercostal neuritis. It is important to note that the patient did not report any symptoms other than pain in the left hip, making femoral head necrosis the most likely diagnosis. Analgesic and neuropathy nutrition were prescribed for the right costal and back pain but the effects were not adequate, so the neurology department was contacted for consultation to assist in controlling the intercostal pain. Following this consultation, non-steroidal anti-inflammatory drugs were prescribed to relieve the patient’s intercostal pain symptoms.

Upon admission, we followed a progressive approach, starting with analgesic and osteo-stimulating drug therapy. However, the patient’s hip pain symptoms were still not well alleviated. After rigorous contemplation and discussion, a surgical decision was finally made to preserve the patient's femoral head. Pre-operative computerized tomography of the pelvis showed bilateral femoral head necrosis, however, the patient consented only to surgery on the left side, as the right side was asymptomatic. The left femoral head necrotic tissue was biopsied for further pathological examination as a routine part. No intra-operative complications occurred. Post-operatively, the patient was given anti-inflammatory analgesic therapy. The patient reported no abnormal discomfort except for occasional pain in the surgical wound. The pathological examination result of the left femoral head necrotic tissue showed metastatic mucinous adenocarcinoma. Immunohistochemical staining showed cancer cells that were CK20, CDX2, and PCK positive and CK7, PSAP, and TTF-1 negative. Once bone metastasis was diagnosed, the patient underwent ultrasonography of the liver and bile ducts, pancreas, spleen, retroperitoneal space as well as testing of the complete set of tumor markers. The ultrasound results were normal with the exception of unrelated intrahepatic bile duct stones or calcification. Tumor marker testing levels were as follows: serum levels of PSA (0.55 μg/L), CA199 < 2.0 U/ml, CA153 (9.4 U/ml), CA125 (15.6 U/ml), α-fetoprotein (7.0 μg/L), SCCA (0.9 ng/ml), and free PSA (0.16 μg/L) were within the normal range. However, serum levels of CEA (218.4 μg/L), ferritin (782.5 μg/L) and CYFRA21-1 (45.08 mg/ml) were increased.

The patient then underwent full-body bone scintigraphy and abdominal enhanced CT. SPECT bone imaging showed irregular bone metabolism on multiple ribs bilaterally and on part of the thoracic vertebrae and 5th lumbar vertebra. In addition, abnormally active bone metabolism was noted in the upper left femur, speculated to be due to postoperative changes. The bone metabolism in the upper segment of the right femur was slightly active and uneven, indicating possible ischemia. No obvious abnormalities were noted in the bone metabolism of the rest of the body. The results of CT enhanced scanning showed small cysts in the right lobe of the liver and right kidney with no enlarged retroperitoneal lymph nodes. Fractures were noted on the 7th thoracic vertebrae, 3rd lumbar vertebrae, and right 5th rib. The patient and his family agreed to be transferred to the tumor center for further treatment.

**Figure 2:**
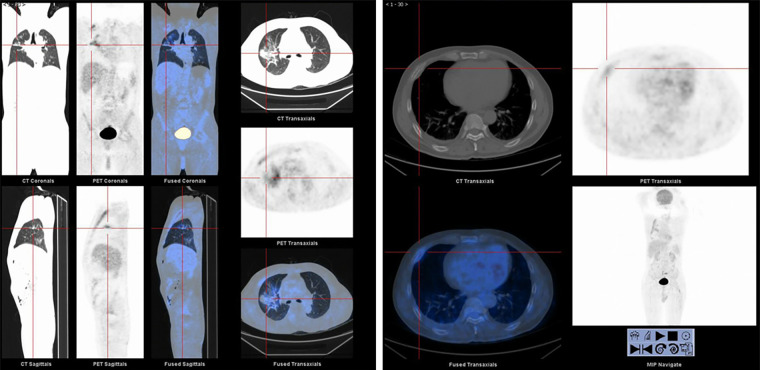
PET-CT images of primary mucinous lung adenocarcinoma with rib metastases.

Once admitted to the tumor ward, the patient first underwent Positron Emission-Computed Tomography (PET-CT) which showed that the metabolism of the anterior part of the right 4th rib was abnormally increased and the nodules and clumps of the upper lobe of the right lung had increased metabolism (Figure 2). On the second day of admission to the tumor ward, the patient developed numbness in both lower extremities. At this point in time, examination showed that the patient had a right lower limb muscle strength of grade II and left lower limb muscle strength of grade III, accompanied by weakened sensation. Based on this observation, the patient was diagnosed with a high possibility of bone metastasis from primary lung cancer, accompanied by spinal cord compression, with poor prognosis and a very short survival time. The patient was given palliative treatment, and MRI examinations of the thoracolumbar vertebrae and pelvis were considered. On the fourth day of admission to the tumor ward, the patient developed paraplegia of the lower limbs bilaterally with grade 0 muscle strength, and rib tenderness in the right anterior chest. After evaluation, the doctors found that the lesions in the high metabolic site of the patient’s lungs were too close to large blood vessels of the lungs, hence high risk of vessel injury and massive bleeding related to puncture prevented a possible biopsy. After optimization of the radiotherapy plan the patient underwent radiotherapy for the thoracic (T3–4) and lumbar (L6–8) vertebrae.

**Figure 3:**
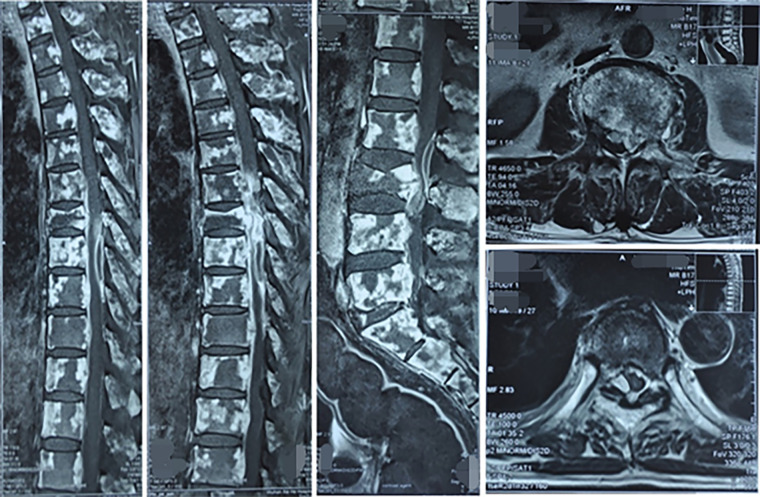
MRI images of vertebral bone metastases and spinal cord compression in primary lung cancer.

The MRI examination showed diffuse abnormalities in the cervical, thoracic and lumbosacral vertebral bodies and their accessory processes, pelvic bones and bone marrow of the upper femurs on both sides, with high possibility of bone metastases. Vertebral compression and pathological fractures in the 7th thoracic vertebrae were also noted. Last, multiple abnormal fusiform signal shadows were noted within the spinal canal and numerous soft tissue masses were noted near the left ilium, all considered to be caused by tumor metastasis (Figure 3). Overall, the tumor had extensive bone metastases, and the paravertebral soft tissue was likely invaded. Therefore, radiotherapy was also required for the paravertebral tissue. However, after 12 rounds of radiotherapy, radiotherapy had to be stopped due to a critical decrease in platelet count.

## Discussion

Pulmonary mucinous adenocarcinoma itself is extremely rare, and usually occurs in people over the age of 60 (12). The clinical symptoms of the disease are varied with no obvious “classic” signs. Most patients are diagnosed when a shadow is seen on the lungs during imaging. The most common clinical symptoms are cough, sputum, chest tightness, and rarely hemoptysis (13). Although the common clinical symptoms of central lung cancer are hemoptysis, most of the central lung cancer have no obvious specific clinical manifestations, which are similar to the clinical manifestations of other lung adenocarcinoma subtypes (14, 15).

The patient in this case report was 53 years old, which is relatively young, and had no other symptoms on the first appearance. His main complaint and reason for visiting orthopedics was pain in the left hip joint. Therefore, initial diagnosis was limited to common conditions causing pain such as femoral head necrosis, tuberculosis or infection. After the patient was hospitalized, right rib and back tenderness were identified during physical examination, originally diagnosed as intercostal neuritis. The true underlying reason however was metastasis of the lung mucinous adenocarcinoma to the spine and ribs. By the time the patient was admitted to the oncology department, his condition was already starting to deteriorate at a fast rate. On the second day of admission to oncology, the patient experienced numbness in both lower limbs, with weakened sensation and decreased muscle strength. On the fourth day, he had paraplegia of both lower limbs, and the muscle strength dropped to grade 0. PET-CT showed that the metabolism of the right upper lobe nodules had increased and there were numerous cluster shadows, suggesting malignant tumor lesions. These results preliminarily established that the lung was the primary tumor lesion. The presenting symptom of the patient was atypical making it initially difficult for the orthopedic surgeons to consider bone metastasis from lung cancer based on the sole symptom of pain in the left hip. This delayed the comprehensive treatment of the tumor. The femoral head preserving treatment we have adopted for the patient avoids the further spread of femoral head tumor metastases. Lung mucinous adenocarcinoma is relatively rare, and bone metastases from lung cancer are difficult to diagnose without clinical symptoms presentation.

We reviewed the related case reports of bone metastasis in mucinous lung adenocarcinoma and found that patients with bone metastasis presented no specific symptoms (Table 1). In this study, the initial manifestation of mucinous lung adenocarcinoma was only symptom with left hip pain. According to the symptom and imaging examinations, it was easily misdiagnosed as femoral head necrosis. In addition, due to the absence of specific symptoms of mucinous lung adenocarcinoma, it is difficult to precisely locate the primary tumor even if mucinous tumor lesion is identified by pathological examination at the metastasis site. Therefore, we hope that this rare case report could encourage clinicians to consider early case of mucinous lung adenocarcinoma when atypical bone metastasis appears.

**Table 1:** Review of case reports of bone metastasis from mucinous lung adenocarcinoma.

Author	Age	Gender	Site of bone metastasis	Presenting symptoms	Histology	Immunohistochemical staining	Abnormal tumor marker
Our study	53	Male	Left hip, spine, ribs and pelvis	Left hip pain	Pulmonary nodules were not pathologically examined because nodules were adjacent to large pulmonary vessels. Pathological examination result of the left femoral head necrotic tissue suggested metastatic mucinous adenocarcinoma.	Left femoral head necrotic tissue: CK20, CDX2 and PCK were positive, CK7, PSAP and TTF-1 were negative	Elevated serum CEA, ferritin and CYFRA21-1
Murai (16)	70	Male	Thoracic vertebrae, ribs, femur and pelvis	Progressive general fatigue was present on admission. Neck pain and right hand numbness 4 months after lung surgery	Pulmonary nodule: Columnar mucinous neoplastic elements floating in large pools of mucus within a fibrous capsule, necrotic tumor cells and dystrophic calcification were observed.	Pulmonary nodule: CK7 was positive, TTF-1 and CK20 were negative	Not reported
Morita (17)	80	Male	Rib	Asymptomatic	Pulmonary nodule: The yellowish-white colloidal nodule was well defined. Microscopically, columnar epithelial cells secreting mucin and floating neoplastic cells were seen.	Pulmonary nodule: CK7, CK20, CDX-2, MUC2 and CEA were positive, Napsin A was partly positive, TTF-1 and SP-A were negative	Elevated serum CEA

## Conclusion

In summary, the case of lung mucinous adenocarcinoma with bone metastases presenting with hip pain as the sole symptom has rarely been reported before. Therefore, by describing this rare case and summing up our experiences, we hope this case report will assist orthopedic surgeons from making a correct diagnosis as early as possible and preventing the delay of treatment of patients with bone metastasis.

## Data Availability

The original contributions presented in the study are included in the article/Supplementary Material, further inquiries can be directed to the corresponding author/s.
